# Altered Feeding Patterns in Rats Exposed to a Palatable Cafeteria Diet: Increased Snacking and Its Implications for Development of Obesity

**DOI:** 10.1371/journal.pone.0060407

**Published:** 2013-04-02

**Authors:** Sarah I. Martire, Nathan Holmes, R. Fred Westbrook, Margaret J. Morris

**Affiliations:** 1 School of Psychology, University of New South Wales, New South Wales, Australia; 2 Department of Pharmacology, School of Medical Sciences, University of New South Wales, New South Wales, Australia; INRA, France

## Abstract

**Background:**

Rats prefer energy-rich foods over chow and eat them to excess. The pattern of eating elicited by this diet is unknown. We used the behavioral satiety sequence to classify an eating bout as a meal or snack and compared the eating patterns of rats fed an energy rich cafeteria diet or chow.

**Methods:**

Eight week old male Sprague Dawley rats were exposed to lab chow or an energy-rich cafeteria diet (plus chow) for 16 weeks. After 5, 10 and 15 weeks, home-cage overnight feeding behavior was recorded. Eating followed by grooming then resting or sleeping was classified as a meal; whereas eating not followed by the full sequence was classified as a snack. Numbers of meals and snacks, their duration, and waiting times between feeding bouts were compared between the two conditions.

**Results:**

Cafeteria-fed rats ate more protein, fat and carbohydrate, consistently ingesting double the energy of chow-fed rats, and were significantly heavier by week 4. Cafeteria-fed rats tended to take multiple snacks between meals and ate fewer meals than chow-fed rats. They also ate more snacks at 5 weeks, were less effective at compensating for snacking by reducing meals, and the number of snacks in the majority of the cafeteria-fed rats was positively related to terminal body weights.

**Conclusions:**

Exposure to a palatable diet had long-term effects on feeding patterns. Rats became overweight because they initially ate more frequently and ultimately ate more of foods with higher energy density. The early increased snacking in young cafeteria-fed rats may represent the establishment of eating habits that promote weight gain.

## Introduction

What people eat is controlled by a number of factors. Sometimes food selection is determined by what is available or affordable, and sometimes by dietary or ethical considerations, religious or cultural practices. However, the major determinant of food selection is hedonics: people select for ingestion what they like and reject what they dislike [1], [2]. One of the factors which determines the hedonic value of a food is its nutrient content. People like foods that are rich in fat, sugar, and protein, select them in preference to foods that are low in these nutrients, and eat more of them [3], [4], [5]. A second factor is variability. People like variety in their diet, select foods that differ in their flavor or texture from those that have been recently consumed, and eat more of them [6]. The modern diet in developed countries has been designed to exploit these sources of liking. This diet is replete with foods that are rich in fat, sugar, and protein, and consists in a wide range of foods that differ in their flavors and textures. Moreover, these foods are readily available, procured with little or no energy expenditure, and are sufficiently cheap as to be affordable by most people in developed countries. The modern diet, its nature, availability and cheapness, together with the modern lifestyle, which is relatively sedentary in terms of leisure activities, transport, and work (when available), are likely to have contributed to the increased prevalence of people who are overweight, even obese, in developed countries [7], [8].

An animal model of these conditions consists in providing laboratory rats with continuous access to a varied diet composed of the same energy rich foods eaten by people. Rats select these foods in preference to laboratory chow, eat excessive amounts relative to their minimal energy expenditure, and, like people in developed countries, become overweight. Such rats double their caloric intake, and develop a marked increase in fat mass, plasma leptin and insulin concentrations [9], [10]. However, little is known about the characteristics of the eating elicited by this diet and whether any such characteristics are related to the increased body weight. For example, rats shifted from standard chow to a varied diet of energy rich foods could continue to eat a similar amount as before, simply increasing their body weight as a consequence of the higher calories in these foods compared to chow. Alternatively, such rats could eat larger meals while maintaining meal number (see Rogers & Blundell, [11]) or eat the same sized meals but more frequently. Finally, rats shifted from chow to the modern diet could, like people, snack on the energy rich foods provided, in addition to eating them as part of a meal.

Rats typically exhibit a stereotyped sequence of behaviors, following an eating bout. This pattern, termed the post-prandial satiety or behavioral satiety sequence, consists in the cessation of eating followed by grooming, resting or sleeping [12], [13]. This transition from eating through grooming to resting or sleeping is associated with natural satiation, for example, it is elicited by a caloric load on the gut and the pre-absorptive satiety factors triggered by that load [such as cholecystokinin (CCK)] [14]. We reasoned that the presence or absence of this sequence can be used to discriminate between feeding bouts which produce satiation (a meal) versus those which do not (a snack). We examined whether rats fed the modern western diet differed from those fed standard chow in terms of their distribution of feeding bouts, and, particularly, in terms of bouts which were or were not followed by the full satiety sequence, that is meals or snacks, respectively. Rats in the diet group were provided with commercially available foods (meat pies, biscuits and so on) in addition to standard chow, and feeding behavior of both groups was assessed across one night after 5, 10, and 15 weeks on their respective diets.

## Methods

### Ethics statement

The experimental protocol was approved by the Animal Care and Ethics Committee of the University of New South Wales and was in accordance with the guidelines provided by the Australian National Health and Medical Research Council.

### Subjects

Subjects were 24 experimentally naïve male Sprague Dawley rats obtained from the Animal Resource Centre (Perth, Australia), aged 7–8 weeks and weighing between 240 and 280 g upon arrival. They were housed in plastic boxes (22 cm height ×65 cm length ×40 cm width) with two rats in each box. Rats were housed two per box rather than in individual boxes because of ethical requirements. The boxes were located in a climate controlled room (22°C) on a 12-hr (7.00 am–7.00 pm) light/dark cycle.

### Diet

During the first week, standard lab chow was provided and rats were handled daily. Water was available throughout the experiment. Following this acclimatization, rats were randomly allocated to either standard lab chow (Group Chow) or a high fat cafeteria diet (Group Cafeteria) condition (n = 12 per group). Standard chow provided 11 kJ/g, 12% energy as fat, 20% protein and 65% carbohydrate (Gordon's Specialty Stockfeeds, NSW, Australia). The food items in the cafeteria diet condition were chosen to reflect the enormous variety, palatability and energy density of the modern western diet [9]. They included Meat Pies, Dim Sims (meat wrapped in rice paper), Pasta, Potato Chips, Oats, Dog Food Roll, assorted cakes (including a sponge cake covered in chocolate and coconut, called a lamington) and biscuits (e.g., cookies), chow mixed with lard and condensed milk, as well as standard chow. Chow mixed with lard and condensed milk, as well as standard chow, was always available. These were supplemented by four of the other foods, two of which were taken from those high in protein and/or carbohydrates (Meat Pies, Dim Sims, Oats, Dog Food Roll), and two were taken from those high in fat/sugar (selection from a range of cakes and biscuits). This diet provided an average of 15.3 kJ/g, 32% energy as fat, 14% protein and 60% carbohydrate, in addition to that provided by the standard laboratory chow. The cafeteria diet was presented daily, at 5 pm, and rats in both groups received their food in hoppers located inside their home cages. Energy intake and body weight were measured once per week. The same five foods were presented on the day on which energy intake was measured each week. The amount consumed was the difference between the weight of the food allocated to a cage and that remaining 24 hr later. Energy intake from the food consumed was calculated using the known energy content of each food (kJ/g).

### Feeding

Feeding behavior was recorded from 7 p.m.–7 a.m. on three occasions, during weeks 5, 10 and 15, using high resolution small dome cameras with infrared LEDs suspended above each cage. One rat in each cage had a dorsal identifying mark that enabled behavior of individual rats to be tracked. Each eating bout was characterized as either a meal or a snack. A meal was defined as a feeding episode that was followed by grooming and then resting or sleeping [15]. A snack was defined as a feeding episode followed by grooming, but without the immediately following resting or sleeping behavior. Behaviors were scored in 30 second intervals. For example, if a rat ate and then groomed, but did not rest within 30 seconds following cessation of grooming behavior, then this was classified as a snack. In contrast, if the rat did in fact rest/sleep within 30 seconds following eating and grooming then this was classified as a meal. Thus if the rat ate, groomed, did not sleep; and after 30 seconds or more ate, groomed and did in fact rest/sleep, this was classified as a snack followed by a meal. This 30 second interval was selected as it was the shortest practical interval given the extended 12 hour recording sessions scored manually at each time point. Eating was scored as ingestion or gnawing of food; grooming was scored as licking of the body or cleaning of the face with forepaws as well as scratching of the body and head with hind legs; resting/sleeping was scored as lying down without movement, typically with the head curled to body [16]. A second observer used the same criteria to score several hours (minimum four rats from each group) from each time point. Scores by the experimenter and the second observer were highly correlated (r^2^ = 0.94, r^2^ = 0.93, r^2^ = 0.95 for weeks 5, 10 and 15 respectively).

### Statistical analyses

Data are expressed as mean ± standard error of mean (SEM). A 4-way ANOVA [with factors of group, current bout (meal or snack), previous bout (meal or snack) and time (5, 10 and 15 weeks)] was used to analyze mean waiting times within feeding sequences. Relative frequency of feeding sequences was analyzed using the chi-squared goodness of fit (GOF) test. The relationships between snack number and percentage snacking (snack number over total bout number ×100), as well as percentage snacking and terminal body weight, were analyzed using correlation analyses. Any difference in the strength of a relationship between the two groups was assessed using the Fisher r-to-Z transformation. All remaining data were analyzed using repeated measures ANOVA, controlling the type 1 error rate (α) at 0.05. Energy intake and feeding data used the cage (each of which contained two rats) as the unit of analysis, *F* critical (1, 10) = 4.9, with all other analyses using each rat as the unit, *F* critical (1, 22) = 4.3. Significant interactions were followed-up using post-hoc simple effects analyses. The error rate for multiple comparisons was controlled using Tukey's HSD method.

## Results

### Energy intake and body weight

**Figure 1:**
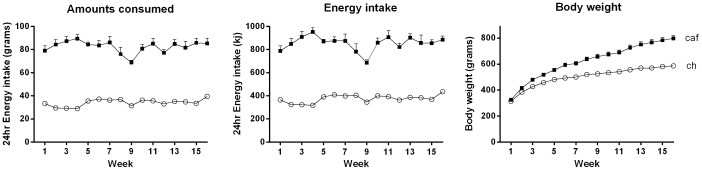
Cafeteria-fed rats consumed more food and energy, and weighed more than chow-fed rats. Mean (±SEM) weekly 24 hr food intake in grams (left), energy intake in kilojoules (middle), and weekly body weight (right), in rats fed either standard lab chow (open circles) or a cafeteria (closed squares) diet for 16 weeks. Data were analyzed using repeated measures ANOVA (energy intake, n = 6 cages per group; body weight, n = 12).

Figure 1 shows mean intake in grams (left) and energy (middle), and mean body weights (right) measured once per week over 16 weeks in rats maintained on either lab chow or cafeteria diet. It is clear that rats fed the cafeteria diet ate more, consumed more energy and showed a greater increase in body weight than those fed chow. The statistical analysis of amounts eaten revealed a significant main effect of group (*F* (1, 10) = 366.2), but no significant effect of time (*F*<2) or time × group interaction (*F* (1, 10) = 4.2). This shows that cafeteria-fed rats ate more than their chow-fed counterparts each week, and that the size of the difference was maintained across the entire study. Similarly, the analysis of energy intake confirmed that there were significant differences between the groups, *F* (1, 10) = 375.1, no effect of time, *F* (1, 10) = 2.99, and no significant interaction between time and groups, *F*<1, indicating that the differences in energy intake between the groups were just as great at the start as at the end of the experiment. The analysis also confirmed that body weight was significantly greater in Group Cafeteria than Chow, *F* (1, 22) = 42.36, increased in both groups across time, *F* (1, 22) = 906.38, and increased more rapidly in Cafeteria than Chow, *F* (1, 22) = 85.09.

**Figure 2:**
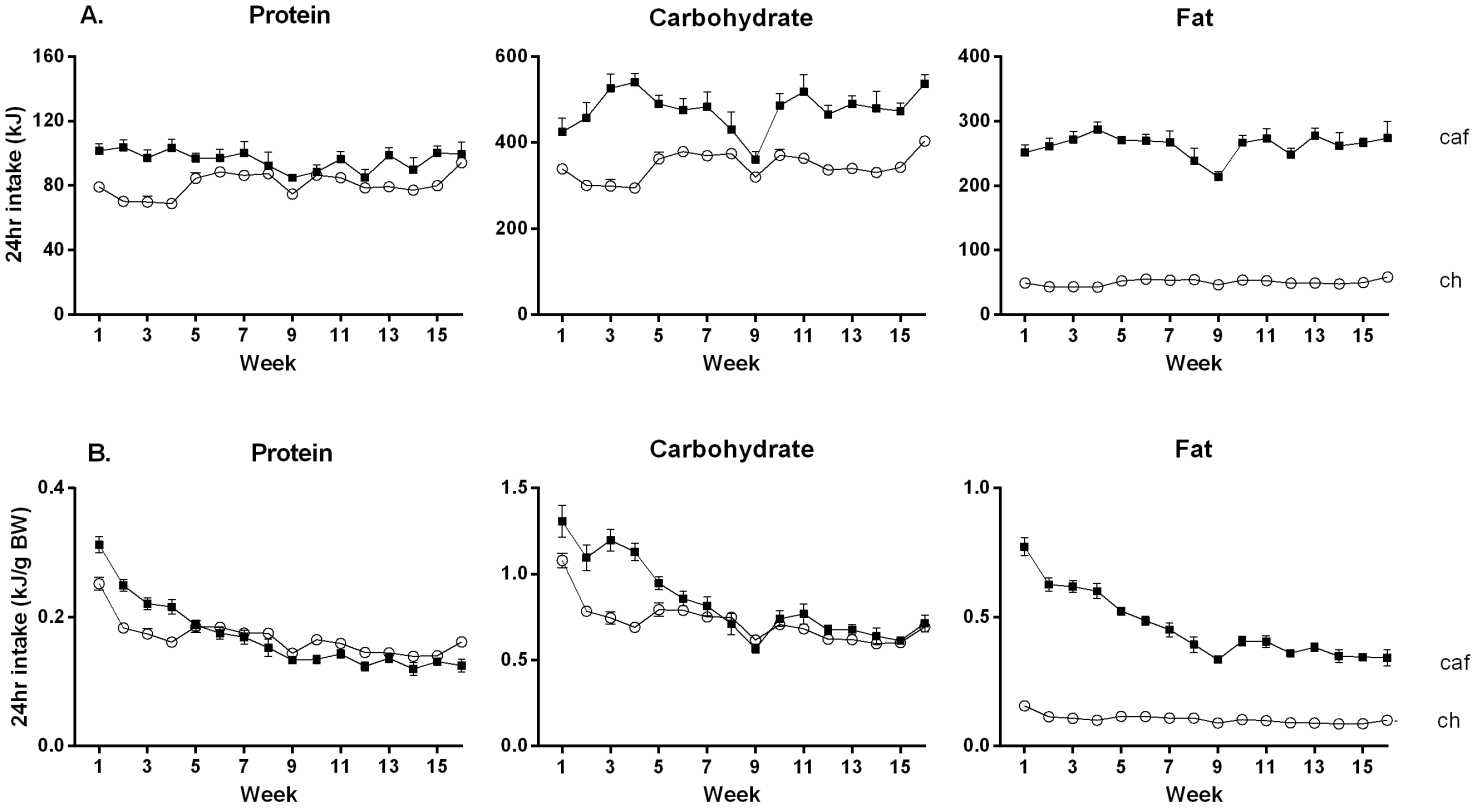
Cafeteria-fed rats persistently consumed more fat, even when adjusted for body weight. Mean (±SEM) weekly macronutrient intake (protein, carbohydrate and fat) in kilojoules (panel A), and in kilojoules adjusted for body weight (panel B) in rats fed either standard lab chow (open circles) or a cafeteria (closed squares) diet for 16 weeks. Data were analyzed using repeated measures ANOVA (n = 6 cages per group).

Figure 2A shows protein, carbohydrate and fat consumption (left, center, and right panels, respectively) measured once per week over 16 weeks. It is clear that Group Cafeteria consumed more of these macronutrients than chow rats and that the differences between their intakes of protein decreased across time but persisted in the case of carbohydrate and fat. The statistical analysis confirmed that protein intake was greater in Group Cafeteria than Group Chow, *F* (1, 10) = 18.32. There was no effect of time on intake, F<1, but there was a significant time × group interaction, *F* (1, 10) = 19.14, indicating that the size of the difference in protein intake between the groups decreased across time. The analysis of carbohydrate intake revealed a significant effect of group, *F* (1, 10) = 57.72, a modest linear trend, *F* (1, 10) = 5.46, and no group × time interaction, *F*<1, confirming that Group Cafeteria persistently ingested more carbohydrate than Group Chow. The evidence for linear trend was partly due to the unexpected decline in carbohydrate intake in week 9. The analysis of the fat intake revealed similar results to those for carbohydrate. Group Cafeteria consumed significantly more than Group Chow, *F* (1, 10) = 777.95, and there were no statistically significant effects of time or time × group interactions, *F*s<1, showing that the greater fat intake in Group Cafeteria persisted over time.

Figure 2B shows protein, carbohydrate and fat intake adjusted for body weight (left, center and right panels, respectively). Inspection of the figure suggests that protein and carbohydrate intake was greater in Group Cafeteria than Chow across the first few weeks but that this difference decreased across subsequent weeks concomitantly with a decreased intake in both groups. Fat intake was markedly greater in Group Cafeteria than Chow. The size of this difference decreased across time, reflecting a decrease in Group Cafeteria and a relative low but stable intake of fat in Group Chow. The statistical analysis supported these impressions. There were no significant differences between the protein intakes overall, *F*<1.0, but there was an effect of time, *F* (1, 10) = 80.90, confirming that intake decreased as body weight increased, and a significant time × group interaction, *F* (1, 10) = 473.96, which reflects the greater initial intake in Group Cafeteria and the decrease in this intake across time to the level in Group Chow. Analysis revealed significantly greater carbohydrate intake in Group Cafeteria than Group Chow, *F* (1, 10) = 16.91, and a significant effect of time, *F* (1, 10) = 176.46, confirming that intake decreased in both groups as body weights increased. There was also a significant time × group interaction, *F* (1, 10) = 26.59, confirming that the size of the difference between the carbohydrate intakes decreased as body weights increased. The analysis of fat intake showed that Group Cafeteria consumed more than Group Chow, *F* (1, 10) = 946.59. Fat intake decreased across time, *F* (1, 10) = 528.81, and there was a significant time × group interaction, *F* (1, 10) = 349.01, which reflects the decreased intake across time in Group Cafeteria and the relatively stable intake in Group Chow.

**Figure 3:**
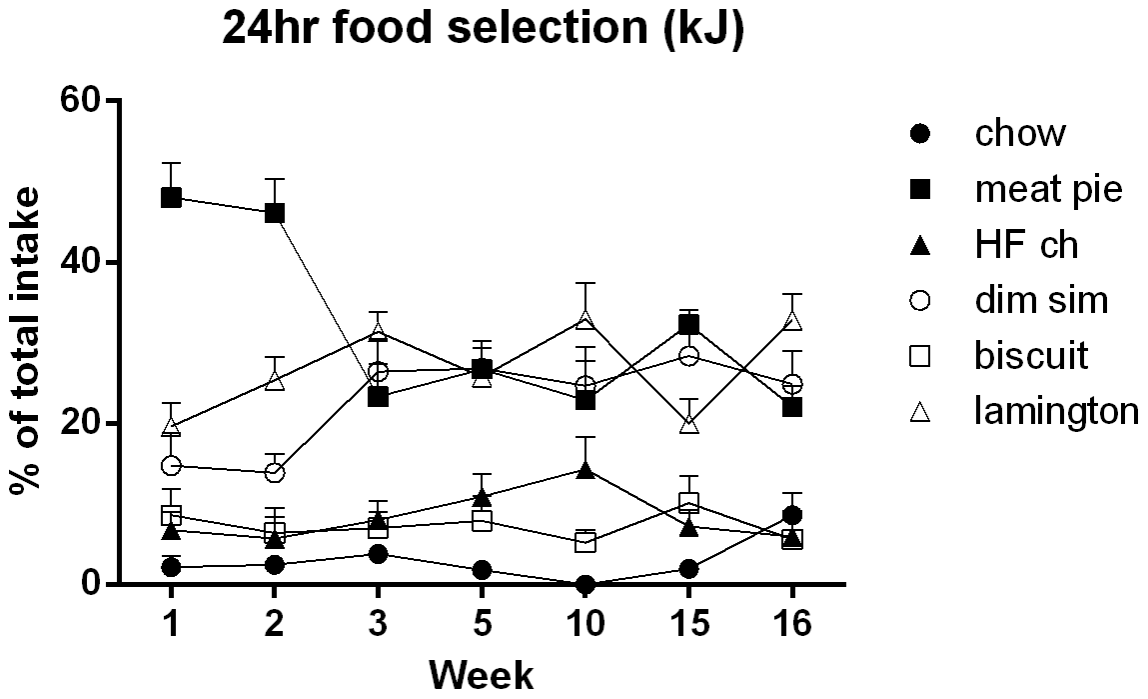
Food selection during 24 hr energy intake measures in cafeteria-fed rats. Proportion of each food type selected as a percentage of total intake (kJ), in cafeteria diet fed rats (chow, closed circles; meat pie, closed squares; high fat chow, closed triangles; dim sim, open circles; biscuit, open squares; lamington, open triangles). Data are shown as mean (±SEM) and were analyzed via repeated measures ANOVA (n = 6 cages per group).

The differences in adjusted protein and carbohydrate intake that were evident across the early weeks decreased as body weights increased in both groups, but the difference in adjusted fat intake persisted across the 16 weeks of exposure to the diet. The decreases in adjusted protein and carbohydrates reflect changes in the foods selected across exposure to the cafeteria diet. Figure 3 shows the proportion contributed by each of the food items in kilojoules to total intake on each of the days when energy intake was assessed. The figure suggests that rats initially selected meat pies, which are high in protein and carbohydrate, in preference to the other foods. This selection decreased concomitantly with an increased selection of Dim Sims and Lamingtons from week 3. Pie, dim sims and lamington intakes remained relatively stable across the remaining weeks, contributing approximately 85% of the total intake. Overall, these foods, as well as the high fat-condensed milk chow, contained 32% energy as fat, in contrast to the standard chow diet whose fat content was 12%. The statistical analysis of the foods consumed by rats on the cafeteria diet confirmed that there were significant differences, (*F* (1, 5) = 30.7), no effects of time (*F*<1), but a significant food × time interaction (*F* (30, 150) = 5.4), which, as noted above, appears to be due to changes in meat pie, dim sims and lamington consumption across time. To verify the source of this interaction, separate repeated measures (linear trend) analyses conducted on the amounts of each food consumed over time showed a significant linear decrease in pie consumption *F* (1, 5) = 20.5, which, from inspection of the figure, was due to a sharp decrease in pie consumption from week 2 to 3, remaining stable thereafter. In contrast, lamington intake showed a significant linear increase from weeks 1 to 3, *F* (1, 5) = 8.2, There was no significant change in consumption of the other foods across time, including dim sims, *F* (1, 5) = 4.7 (*F* critical = 6.6).

Taken together, these results show that Group Cafeteria ate more in grams, had greater energy intake, and gained weight at a faster rate than Group Chow. Moreover, Group Cafeteria consumed more net protein, carbohydrate and fat than Group Chow. When adjusted for body weight, the difference between the groups in fat consumption persisted. Analysis of the foods selected by Group Cafeteria showed that the persistent difference in adjusted fat consumption was due to the fact that this diet was, quite simply, high in fat. Within this high fat diet, Group Cafeteria tended to prefer the foods that were the richest sources of protein (pie and dim sims), suggesting that they may have been selecting foods based on their protein content. However, it must be noted that Group Cafeteria had continuous access to chow whose protein content is high, yet was not selected. Indeed chow was the least preferred of the foods available to Group Cafeteria (making up 5% of total intake), suggesting that protein-seeking alone cannot explain their increased intake. Finally, the fact that there was little change in the foods selected by Group Cafeteria over time (with the exception of a decrease in meat pie and increase in lamington consumption between weeks 1 and 3) means that the proportion of total intake contributed by each macronutrient remained constant over time.

### Microstructure of Feeding

#### Meals

**Figure 4:**
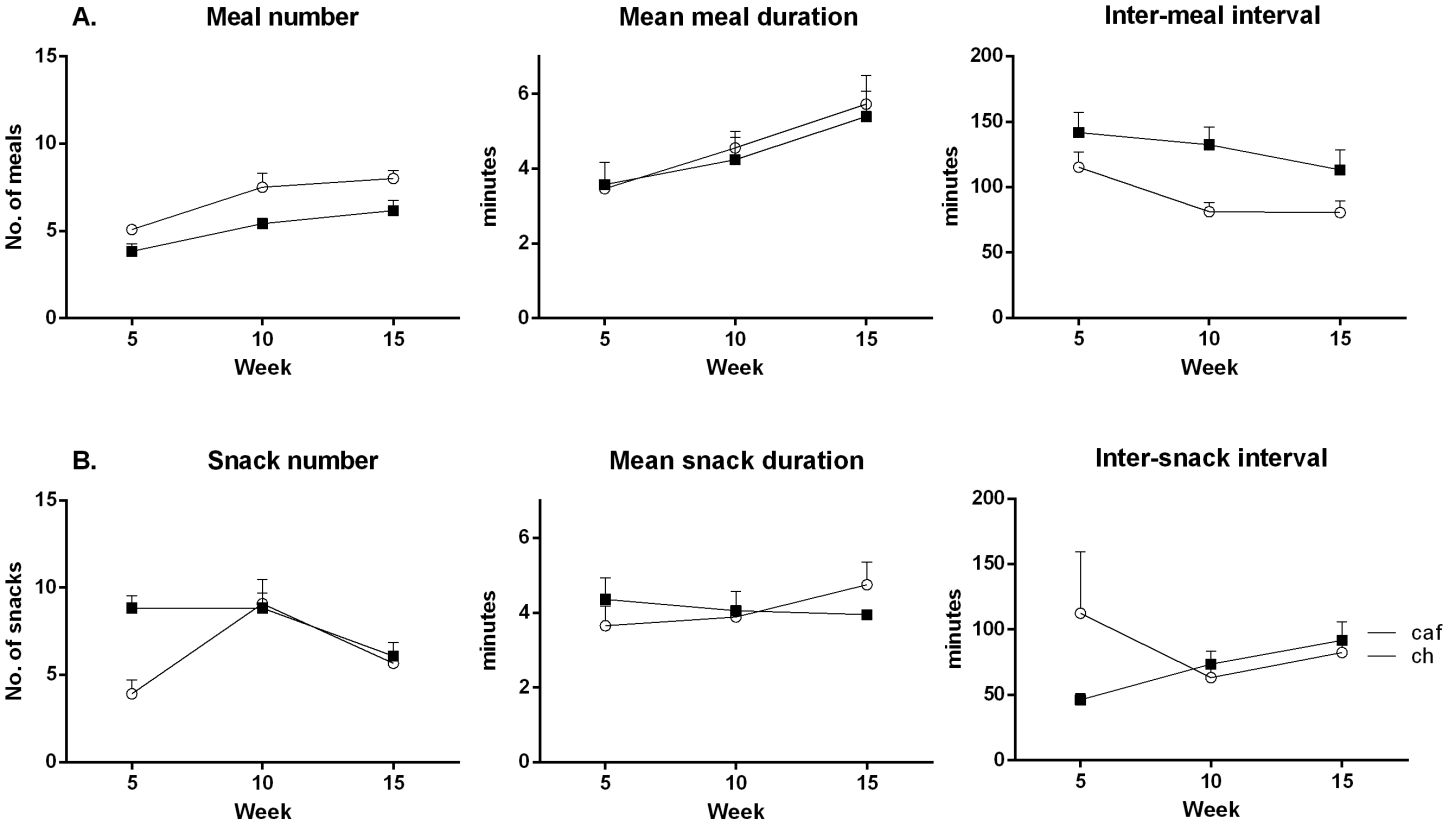
Cafeteria-fed rats consumed consistently fewer meals but more snacks early in diet exposure. Mean (±SEM) number of meals (left), mean meal duration (middle), and mean inter-meal interval (right) per cage (panel A), and mean number of snacks (left), mean snack duration (middle), and mean inter-snack interval (right; panel B), during the dark phase (7 pm–7 am) at 5, 10, and 15 weeks of diet in chow (open circles) or cafeteria (closed squares) fed rats. Data were analyzed via repeated measures ANOVA (n = 6 cages per group).

Figure 4A shows the mean number of meals (left), mean duration of each meal (middle), and mean interval between meals (right) for Groups Cafeteria and Chow over one night in weeks 5, 10 and 15. The statistical analysis of the number of meals confirmed that Group Cafeteria ate fewer meals than Group Chow, *F* (1, 10) = 14.85. Both groups ate more meals across time, (*F* (1, 10) = 23.85) but the time × group interaction was not significant, *F*<1, showing that Group Cafeteria persistently ate fewer meals than Group Chow. The difference in the number of meals was not due to Group Cafeteria spending more time eating during each meal than Group Chow (middle panel). There was no significant difference between the groups in meal duration, F<1. There was a significant effect of time on meal duration (*F* (1, 10) = 18.90), confirming that durations increased across time, but there was no statistically significant group × time interaction (F<1). The right panel shows that rats in Group Cafeteria waited longer between meals than those in Group Chow, *F* (1, 10) = 17.16 (right). The interval between meals increased across time. This increase approached but did not reach a conventional level of significance (*F* (1, 10) = 4.54, and there was no group × time interaction, *F*<1, showing that the differences in inter-meal intervals between the groups persisted across the three time points.

#### Snacks

Figure 4B shows the mean number of snacks (left), mean duration of snacks (middle) and mean interval (right) between snacks at weeks 5, 10 and 15. Inspection of the figures suggests that at the 5 week time point Group Cafeteria snacked more than Group Chow, but this difference between the groups was absent at 10 and 15 weeks. The difference between the number of snacks eaten by the two groups approached but did not reach a conventional level of significance, *F* (1, 10) = 4.84, and there was no effect of time, *F*<1. However, there was a significant time × group interaction, *F* (1, 10) = 8.53, which as noted above, is due to the fact that rats in Group Cafeteria ate more snacks than those in Group Chow at week 5, *F* (1, 10) = 21.30, but not at weeks 10 and 15, *F*s<1. There was no difference between the groups in the duration of snacks (*F*<2), and there was no effect of time or time × group interaction (*F*s<2). Group Cafeteria appeared to have shorter intervals between snacks than Group Chow at 5 weeks but not at the later time points. However, statistical analysis failed to reveal a significant difference between the groups, an effect of time, or a group × time interaction, (*F*s<2.5; right).

#### Total Eating Time

**Figure 5:**
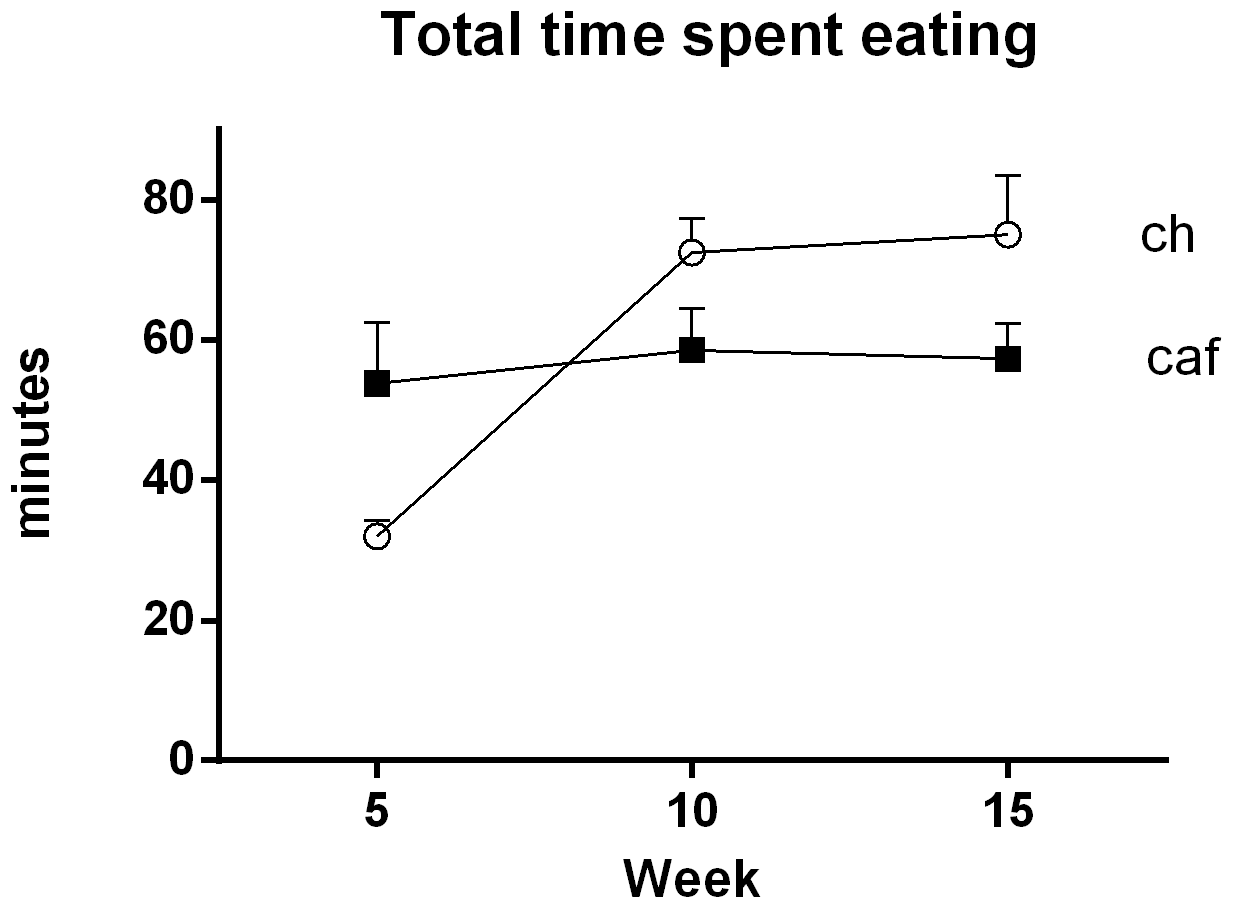
Cafeteria-fed rats spend more total time eating early but not later in diet exposure. Mean (±SEM) total time spent eating during the dark phase (7 pm–7 am) in rats fed either standard lab chow (open circles) or a cafeteria (closed squares) diet at 5, 10, and 15 weeks. Data were analyzed using repeated measures ANOVA (n = 6 cages per group).

Figure 5 shows the total time spent eating at each of the three time points for Groups Cafeteria and Chow. This time was relatively stable across weeks 5, 10 and 15 in Group Cafeteria but increased across these assessments in Group Chow. The statistical analysis failed to reveal an overall difference between the groups, *F*<1. However, there was a significant effect of time, *F* (1, 10) = 8.89, and a significant time × group interaction, *F* (1, 10) = 6.42. Post-hoc analyses of simple effects failed to detect significant differences between the groups at any time point (largest *F* (1, 10) = 5.93). This suggests that variance in both group and time contributed to the interaction between these factors.

Taken together, these results show that Group Cafeteria consistently ate fewer meals than Group Chow but ate more snacks, at least initially. These differences in the numbers of meals and snacks eaten were not due to differences in the amounts of time spent eating. Rather, Group Cafeteria waited longer between meals. At 5 weeks, the longer waiting time between meals can be partly explained by the fact that Group Cafeteria snacked more. However, Group Cafeteria continued to wait longer between meals at the 10 and 15 time points despite similar snacking behavior as Group Chow. Thus, the fact that Group Cafeteria continued to wait longer between meals than Group Chow at the later time points must be due to other factors.

#### Relative frequency of specific sequences of meals and snacks

**Figure 6:**
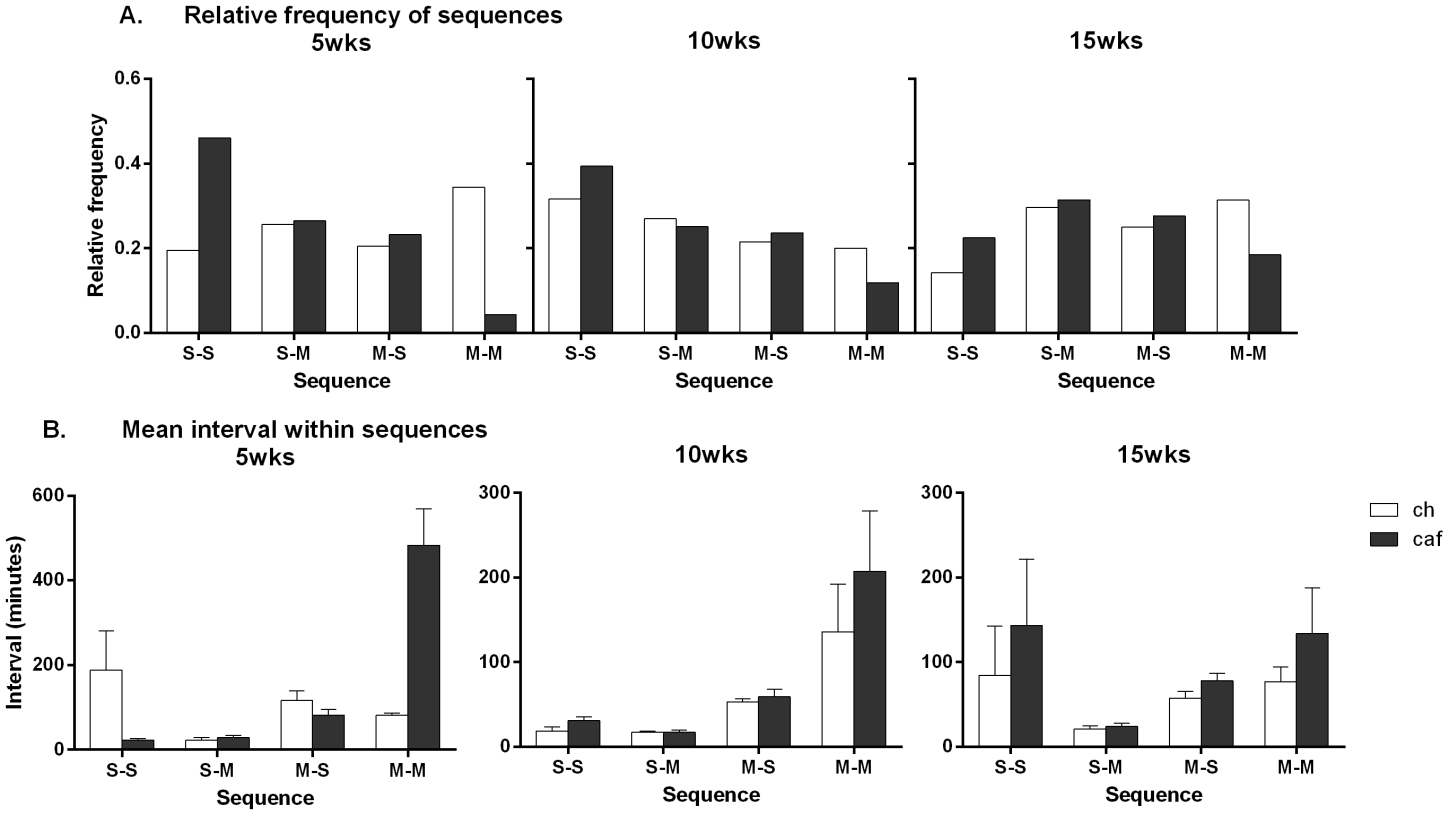
Cafeteria-fed rats are more likely to eat consecutive snacks between meals. Mean relative frequency of different feeding sequences at 5 (left), 10 (middle) and 15 (right) weeks; panel A), S-S: snack followed by snack, S-M: snack followed by meal, M-S: meal followed by a snack, M-M: meal followed by a meal. Panel B shows mean (±SEM) intervals between elements of a sequence (e.g. the average interval between a snack followed by a snack) during the dark phase (7 pm–7 am) following 5 (left), 10 (middle), and 15 (right) weeks of diet, in chow (open bars) and cafeteria (closed bars) fed rats. Data were analyzed via the Chi-squared goodness of fit test (relative frequency of sequences) and repeated measures ANOVA (intervals within sequences).

To determine how waiting times between feeding bouts were related to the prior feeding episode (i.e., whether it was a meal or a snack), meal and snack data for Groups Chow and Cafeteria at the 5, 10 and 15 week time points were classified into sequences that consisted in a snack followed by a snack (S-S), snack followed by a meal (S-M), a meal followed by a snack (M-S), and a meal followed by a meal (M-M). Figure 6A shows the relative frequency of each sequence at 5 (left), 10 (middle) and 15 (right) weeks in Groups Chow and Cafeteria. The figure suggests that at the 5 week time point (left panel) Group Cafeteria had a larger proportion of S-S sequences than Group Chow. In contrast, Group Chow appeared to have a larger proportion of M-M sequences than Group Cafeteria. These differences decreased at the later time points. The chi-squared GOF test confirmed statistically significant differences in the relative frequency of S-S sequences (χ^2^ (1) = 52.2, *p*<0.0001), and M-M sequences (χ^2^ (1) = 36.9, *p*<0.0001) between Group Chow and Group Cafeteria at the 5 week time point. There were no statistically significant differences between the groups in S-M and M-S sequences. These trends in relative frequency of feeding sequences were also present at the 10 and 15 week time points, but there were no statistically significant differences between the two groups at either time point (largest χ^2^ (1) = 0.6, *p*>0.05).

#### Mean waiting times within a sequence

We examined whether the mean waiting time to a snack or a meal was related to the identity of the previous feeding bout, and whether this contributed to the previously described group differences in waiting times between meals. Mean waiting times within each sequence at the 5, 10, and 15 week time points are shown in Figure 6B. Inspection of the figure suggests that, at each of the time points, average waiting times to a feeding bout (regardless of whether it was a meal or snack) were longer if the preceding bout was a meal than if it was a snack, supporting the use of the behavioral satiety sequence to identify a feeding bout as a meal, that is, a feeding bout which produced satiety. At the 5 week time point, having had a snack, Group Cafeteria tended to have shorter waiting times to the next snack than Group Chow; however, having had a meal, Group Cafeteria tended to wait longer to the next meal than Group Chow. These differences in waiting times between the elements of a feeding sequence appeared to have decreased at the 10 and 15 week time points. A 4-way ANOVA [with factors of group, current bout (meal or snack), previous bout (meal or snack) and time (5, 10 and 15 weeks)] revealed significant main effects of previous bout (*F* (1, 22) = 14.0) and time (*F* (2, 44) = 6.9), confirming that waiting times were longer following a meal compared to a snack, and that average waiting times between elements of a sequence decreased over the three time points. There were significant interactions between group × current bout × previous bout × time (*F* (2, 44) = 7.1), current bout × time × group (*F* (2, 44) = 6.7), previous bout × time × group (F (2, 44) = 8.0), current bout × previous bout × time (*F* (2, 44) = 12.0), time × current bout (*F* (2, 44) = 3.6) and time × previous bout (*F* (2, 44) = 11.6; *p*s<0.05).

To determine the source of these interactions, we conducted separate repeated measures analyses over time for each of the feeding sequences. Analysis of average waiting times to a meal given a snack (S-M) and to a snack given a meal (M-S) showed that the main effects of group and time, as well as their interaction were not significant (*F*s<4). Analysis of average waiting times to a snack given a snack (S-S) showed that the main effects of group and time were not significant (*F*s<1). However, the group × time interaction approached significance (*F* (1, 22) = 4.2), suggesting that the groups differed at the 5 week time point but not thereafter. In contrast, analysis of average waiting times to a meal given a meal (M-M) revealed clear effects of group (*F* (1, 22) = 13.8) and time (*F* (1, 22) = 15.9), as well as an interaction between these factors (*F* (1, 22) = 14.3). Again, this interaction is due to a clear difference between the groups at 5 weeks which decreased at the later time points. (*F* critical = 4.3).

These results show that the sequences of feeding bouts at the 5 week time point differed between the two groups of rats. Group Cafeteria was far more likely to have a snack followed by another snack than was Group Chow, whereas Group Chow was more likely to have a meal followed by another meal. At the 5 week time point, the groups also differed in the waiting times between snacks and meals that occurred consecutively, with Group Cafeteria tending to have shorter waiting times between consecutive snacks but longer waiting times between consecutive meals. The differences between Groups Cafeteria and Chow in feeding sequences and waiting times between sequence elements were significantly diminished at the later time points.

Importantly, the use of the behavioral satiety sequence to classify feeding bouts as either meals or snacks was validated through examination of waiting times between the elements of a sequence. Specifically, both cafeteria and chow fed rats had a longer average waiting time to a bout (meal or snack) when that bout was preceded by a meal as opposed to a snack. This is consistent with the notion that, as distinct from snacks, feeding bouts characterized by a complete behavioral satiety sequence were those that lead to satiety (i.e., meals). We used an interval equal to or greater than 30 seconds to identify two distinct eating bouts whereas an interval of less than 30 seconds between two eating bouts was classified as a single bout. Thus, if the interval between a snack and the next feeding bout (regardless of whether it was a snack or meal) was only slightly greater than 30 seconds, it could be argued that bouts should not be classified as consecutive snacks (S-S) or a snack followed by a meal (S-M), but rather as a single snack or meal. However, contrary to this argument, inspection of the average waiting times between a snack and the next feeding bout suggested that snacks tended to occur in relative isolation for both groups. Across all time points, the minimum average interval between a snack and the next feeding bout was 17.0 minutes for Group Chow (S-M interval in week 10) and 17.6 minutes for Group Cafeteria (S-M interval in week 10). Moreover, the fact that these intervals are on a scale of minutes (as opposed to seconds) means that use of the 30 seconds criterion to identify one feeding bout from the next is unlikely to have differentially affected Groups Chow and Cafeteria – i.e., the observed differences in snacks and meals between the two groups are not an artifact of the 30 seconds criterion used in the classification of separate feeding bouts.

#### The relationship between snacking and weight gain

Differences in snacking between the two groups during the early stages of diet exposure may have contributed to the differences in their weight gain. One possibility is that snacking is directly related to weight gain such that rats in either group who snacked more gained weight more rapidly. Alternatively, rats who snacked more may have compensated by reducing the number and/or duration of meals consumed, thereby gaining weight more slowly. Before examining how snacking affected weight gain, we first examined whether rats in either group were in fact able to compensate for energy obtained through snacking. We specifically asked whether rats who ate a large number of snacks compensated by reducing the number of meals which they ate. If rats did in fact compensate by reducing number of meals, this would be reflected in the relationship between the numbers of snacks that they consumed and the percentage of bouts classified as a snack (i.e., a reduction in meal number must imply an increase in percentage snacking).

**Figure 7:**
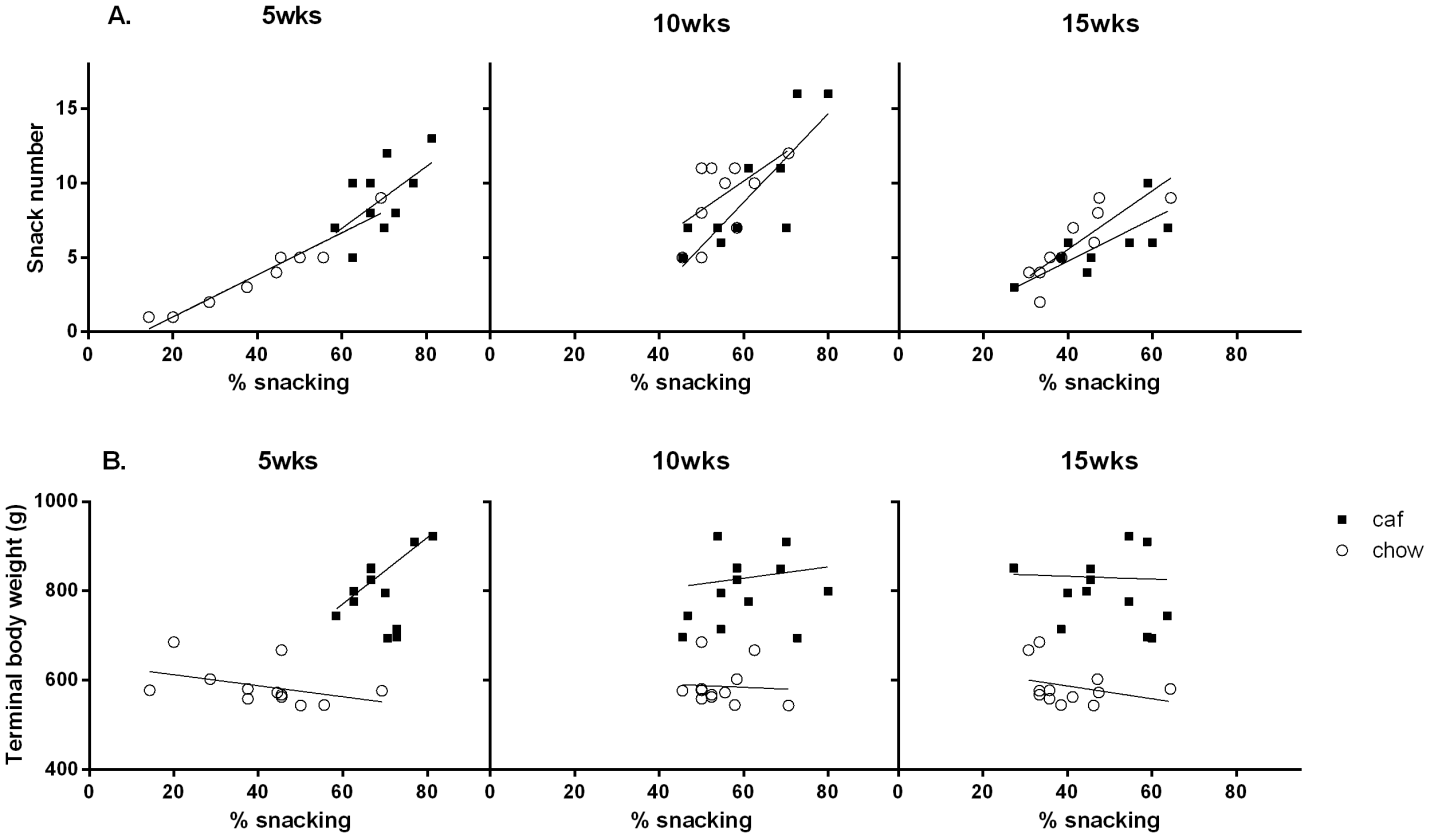
Percentage snacking at 5 weeks correlated with terminal body weights of cafeteria-fed rats. Mean snack number plotted against mean percentage snacking (panel A), and terminal body weight plotted against mean percentage snacking (panel B) in chow (open circle) and cafeteria-fed (closed square) rats at 5 (left), 10 (middle) and 15 (right) weeks of diet. Outliers in the cafeteria-fed group (the three lightest rats in this group) were excluded from the analyses. Data were analyzed using correlation analysis.

Figure 7A shows the relationship between numbers of snacks and percentage snacking in Groups Chow and Cafeteria after 5 (left), 10 (middle) and 15 weeks (right) on the respective diets. As noted above, Group Cafeteria snacked more (in both number and percentage terms) than Group Chow at 5 weeks, but not thereafter. Moreover, the relationship between snack number and snack percentage differed between the two groups at 5 weeks, but not thereafter. This was confirmed in the statistical analysis. After 5 weeks, snack number was significantly correlated with percentage snacking in both groups (r^2^ = 0.93 and 0.36 for Groups Chow and Cafeteria, respectively, *p*s<0.05), indicating that both groups showed some degree of compensation for their snacking behavior. The significance of the difference between correlation coefficients for cafeteria- and chow-fed rats was assessed using the Fisher r-to-z transformation. Critically, this revealed that the relationship between snack number and percentage snacking was significantly stronger in Group Chow (*z* = 2.78, *p*<0.01), suggesting that these rats more effectively compensated for their snacking than those in Group Cafeteria. After 10 and 15 weeks, snack number remained significantly correlated with percentage snacking in both groups, with the exception of Group Chow at 10 weeks which approached significance (10 weeks, r^2^ = 0.31 *p*<0.06, and 0.68 *p*<0.01, for Groups Chow and Cafeteria, respectively; 15 weeks, r^2^ = 0.73 and 0.54 for Groups Chow and Cafeteria, respectively; *p*s<0.01:). Critically, the earlier difference in the strength of this relationship between the two groups was no longer evident (larger *z* = 1.15, *p*>0.05).

#### How does terminal body weight relate to snacking at 5, 10 and 15 weeks?

Next we examined how variations in compensation related to total weight gain. Figure 7B shows the relationship between terminal body weight and percentage snacking at 5 (left), 10 (middle) and 15 (right) weeks. In Group Chow, there appears to be no relationship between percentage snacking at any of the time points and terminal body weight. In contrast, in Group Cafeteria, inspection of the Figure suggests that there was in fact a relationship between percentage snacking at 5 weeks and terminal body weight, but not thereafter. What is apparent in the Figure however, is the cluster of three data points that represent the lightest rats in that group. When these three rats are excluded from the analysis (on the grounds they did not gain weight in the same manner as the other rats in the group), there is a clear linear relationship between percentage snacking and terminal body weight in this group. The statistical analysis showed that terminal body weight did not correlate with percentage snacking at any time point in Group Chow (largest r^2^ = 0.17, *p*>0.05). In Group Cafeteria however, terminal body weight showed a positive linear correlation with percentage snacking at 5 weeks (r^2^ = 0.82, *p*<0.01); but did not correlate with percentage snacking at any other time (larger r^2^ = 0.35, *p*>0.05).

All rats snacked. All rats showed some degree of compensation for this snacking by reducing meal number. After 5 weeks, Group Chow was more effective at compensating for snacking than Group Cafeteria. The two groups showed similar compensation for increased snacking after 10 and 15 weeks. Critically, there was a clear relationship between terminal body weights and percentage snacking after 5 weeks in Group Cafeteria: Those rats who snacked the most (in percentage terms) were among the heaviest in this group, thus, snacking in this group was associated with substantial weight gain.

## Discussion

This experiment has confirmed that laboratory rats select the energy rich foods eaten by people in preference to standard chow, eat these foods to excess and become overweight. Rats exposed to this cafeteria diet increased their body weight more than those fed chow after four weeks on their respective diets, continued to increase their body weights faster than chow fed rats, and had increased their body weight by approximately 270% after 16 weeks on the diet relative to the gain of 170% by rats fed chow. Rats on the cafeteria diet obtained double the energy of rats on the chow diet, initially obtained more protein and carbohydrate, and persistently consumed more fat, both net and per gram body weight. The initial high intake of fat presumably reflects its palatability. However, persistently high fat intakes – even when energy requirement is exceeded – may be due to other factors. For example, dietary fat impairs both oral and intestinal nutrient sensing [17], [18], [19], which would reduce detection of excessive fat intake, leading to insulin insensitivity [20]. Hence, rats may have continued to eat excessive amounts of the high fat foods regardless of rapid weight gain, and despite the continuous availability of chow, complete in macronutrient requirements, yet the least likely to be selected (5% of total intake). There was some evidence that cafeteria-fed rats selected foods richest in protein, at least initially. Indeed, when the richest source of protein (meat pies) was removed from the energy intake data, early (4 weeks) differences seen in body weight adjusted protein intake between the groups disappear (data not shown). Foods rich in protein may have been selected because this nutrient is more effective in producing postprandial satiety than carbohydrate and fat [21], [22]. However, as mentioned earlier, this does not explain why rats did not select chow, rich in protein relative to the cafeteria style foods.

The cafeteria diet could lead to excessive weight gain simply because the foods that comprise the diet are more energy dense. Alternatively, that diet could encourage more frequent eating, eating of larger portions, or some combination of these factors. The results were clear. Cafeteria-fed rats ate more than chow-fed rats, the foods they ate were more energy dense, and therefore, they gained excessive weight. These gross differences in amounts eaten and energy intake were accompanied by marked differences in eating patterns. We used the behavioral satiety sequence to identify an eating bout as a meal and the absence of the full sequence as a snack. Using this classification, we found that cafeteria-fed rats snacked more frequently than chow-fed rats during the early (week 5) but not later (weeks 10 and 15) stages of the diet. Early snacking in cafeteria-fed rats was characterized by the fact that, having snacked, these rats were far more likely to snack again, and to do so after relatively little time had elapsed. In contrast, cafeteria-fed rats ate fewer meals than chow-fed rats across all time points in the study.

These eating trends suggest that, in the early weeks, excessive energy intake in cafeteria-fed rats may have been partly due to the fact that the cafeteria diet encouraged more frequent snacking. However, over-eating and excessive energy intake persisted across later stages of the diet when, as noted, if anything, cafeteria-fed rats spent less time eating than chow-fed rats. Thus, over-eating and excessive energy intake in these rats later in diet exposure was not due to the fact that they ate more frequently. Moreover, the differences between the two groups in amounts eaten and energy intake persisted even when adjusted for body weights, suggesting that over-eating and excessive energy intake in cafeteria-fed rats were not simply due to the fact that they were heavier (data not shown). Instead, these results imply that cafeteria-fed rats ate larger portions of the foods that they had become accustomed to eating early in the diet; therefore, their energy intake remained excessive and they gained excessive weight. It is worth noting however that, across weeks, there were no changes in the amount of time that cafeteria-fed rats spent eating, or in the amounts of food that they consumed (in both grams and kilojoules). Therefore, the fact that cafeteria- and chow-fed rats had different portion sizes was not due to an increase in portion size in the former group. Rather, chow-fed rats spent more time eating the same amount of food (in grams and kilojoules) across weeks of the diet, implying that portion size specifically decreased in this group. This result implies that the nature of the cafeteria diet was such that rats did not appropriately decrease portion sizes as they gained weight.

The overall picture that emerges from these findings is that early snacking may be a critical determinant of weight gain in cafeteria-fed rats. Early weight gain in these rats may have been excessive because they failed to reduce meal numbers in compensation for energy obtained through snacking. We reasoned that rats which failed to compensate for energy obtained through snacking would have more meals relative to their number of snacks, and therefore, snacking would make up a smaller percentage of their total eating behavior. In this respect, rats in both groups showed some degree of compensation. However, at the early time point, the relationship between snack numbers and percentage snacking was weaker in cafeteria-fed rats compared to chow-fed rats. This reduced ability to compensate at the early time point was related to terminal body weights. Those rats for whom snacking made up a large percentage of eating behavior were among the heaviest of the cafeteria-fed rats. Critically, there were no significant relationships between percentage snacking and terminal body weights at either of the later time points, suggesting that it was specifically early snacking behavior that set rats on a path which led terminally high body weights.

It is clear that a cafeteria diet initially encourages snacking on energy rich foods which are eaten to excess. Why do cafeteria diets encourage snacking? One explanation for this may be that the foods selected as snacks by the cafeteria group at 5 weeks were less likely to lead to satiety than chow. The high fat content of the cafeteria foods in particular would have contributed to this lack of satiation. For example, high fat diets often result in lower postprandial suppression of ghrelin, which acts as a potent hunger signal, relative to carbohydrates and protein [23], [24]. Variety in the cafeteria diet must also be considered. The range of foods available would have reduced the effect of sensory-specific satiety, thereby increasing intake [25], [26]. Specifically, rats offered the cafeteria diet may switch between foods, maintaining palatability and increasing the likelihood of consecutive bouts without rest/sleep, that is, of consecutive snacks. In contrast, rats fed chow may have terminated eating and rested/slept once sensory-specific satiety occurred. Any such effect of variety, however, does not explain why the increase in consecutive snacking seen in cafeteria-fed rats at 5 weeks was no longer evident at 10 and 15 weeks. Perhaps the effect was no longer seen because the foods presented had become familiar and/or less hedonically attractive.

In a previous study, Rogers and Blundell [11] examined feeding patterns in rats exposed to a cafeteria diet. They found that these rats initially ate more meals than chow-fed rats (where a meal was defined retrospectively as at least 1 minute of eating followed by an interval of at least 15 minutes without eating), but that this difference declined across the course of the study. In contrast, rats on the cafeteria diet ate larger meals than chow-fed rats across the entire duration of the study. These findings seemingly stand in contrast to those obtained in the present study where Group Cafeteria ate persistently fewer meals than Group Chow. However, there are two important differences between the present study and that of Rogers and Blundell [11]. First, the cafeteria diet in the earlier study consisted of chow, white bread crumbs and chocolate flakes, whereas the diet used here contained a wider range of foods; a range intended to model the variety provided by the diet in developed countries. Second, the differences between the meal patterns in the two studies likely relate to the differences in how a meal is defined [eating bout of at least one minute followed by the absence of eating for at least 15 minutes versus an eating bout followed by grooming and resting/sleeping].

Several aspects of the present findings are mirrored in people where obesity has been associated with both increased snacking [27], [28] and increased portion sizes [29], [30]. Both of these factors accompanied the development of obesity in the present study in a manner that depended on experience with the diet: frequent snacking resulting in more total bouts was evident early in the diet, and by inference, larger portion sizes were consumed later in the diet. Early and frequent snacking may be especially critical to the development of obesity. Excess energy, and therefore, weight gain may reflect a failure to compensate for the calories obtained through snacking across initial exposure to an energy rich diet, and consumption of larger portions across later exposure to that diet. There is evidence that both of these factors contribute to weight gain and obesity in people [31], [32], [33], [34], [35].

The early increase in snacking and persistent reduction in meals observed here is characteristic of eating patterns in adolescents (prior to obesity). Adolescents tend to snack throughout the day, skip meals [36], and snack on energy-rich foods including fast foods [37]. Snacking in young adults has increased concurrently with the rise in obesity [38], supporting the link between the modern diet and changes in eating patterns. Thus early adulthood may represent a sensitive period in which eating patterns that promote weight gain are established.

The present experiment is the first to record the eating patterns of rats free to consume the energy rich foods eaten by people, and to use the behavioral satiety sequence as a way of classifying an eating bout as a meal or snack. The results are significant in two respects. First, they have important implications for dieting. Current weight loss treatments are only marginally effective in the long-term. Knowledge regarding eating patterns associated with excessive intake may assist in weight loss treatment programs, as well as in detecting individuals at risk for obesity. Second, increased snacking early in the diet period was related to greater terminal body weights in those consuming the cafeteria diet. This suggests that early and frequent consumption of palatable foods may interfere with satiety signals, and thus induce eating patterns that promote overconsumption throughout adulthood.
